# Nitrogen Advanced Treatment of Urban Sewage by Denitrification Deep-Bed Filter: Removal Performance and Metabolic Pathway

**DOI:** 10.3389/fmicb.2021.811697

**Published:** 2022-01-26

**Authors:** Xiao Huang, Yixiao Xing, Hongjie Wang, Zhongyi Dai, Tiantian Chen

**Affiliations:** ^1^Jiangsu Key Laboratory of Atmospheric Environment Monitoring and Pollution Control, Collaborative Innovation Center of Atmospheric Environment and Equipment Technology, School of Environmental Science and Engineering, Nanjing University of Information Science and Technology, Nanjing, China; ^2^Shenzhen Key Laboratory of Water Resource Utilization and Environmental Pollution Control, School of Civil and Environmental Engineering, Harbin Institute of Technology, Shenzhen, China; ^3^State Key Laboratory of Urban Water Resource and Environment, School of Environment, Harbin Institute of Technology, Harbin, China; ^4^China Municipal Engineering Central South Design and Research Institute Co., Ltd., Wuhan, China; ^5^CAS Key Laboratory of Marine Ecology and Environmental Sciences, Institute of Oceanology, Chinese Academy of Sciences, Qingdao, China

**Keywords:** advanced treatment, denitrification deep-bed filter, conditions optimization, total nitrogen, metabolic pathway

## Abstract

This study aimed to explore the performance of denitrification deep-bed filter (DN-DBF) to treat municipal sewage for meeting a more stringent discharge standard of total nitrogen (TN) (10.0 mg L^–1^). A lab-scale DN-DBF was conducted to optimize operation parameters and reveal the microbiological mechanism for TN removal. The results showed that more than 12.7% TN removal was obtained by adding methanol compared with sodium acetate. The effluent TN concentration reached 6.0–7.0 mg L^–1^ with the optimal influent carbon and nitrogen ratio (C/N) and hydraulic retention time (HRT) (3:1 and 0.25 h). For the nitrogen removal mechanism, *Blastocatellaceae_Subgroup_4 and norank_o_JG30-KF-CM45* were dominant denitrification floras with an abundance of 6–10%. Though large TN was removed at the top layer of DN-DBF, microbial richness and diversity at the middle layer were higher than both ends. However, the relative abundance of nitrite reductase enzymes (EC1.7.2.1) gradually increases as the depth increases; conversely, the relative abundance of nitrous oxide reductase gradually decreased.

## Introduction

Eutrophication, caused by excessive discharged nutrients (nitrogen and phosphorus) from wastewater treatment plants, has become one of the most urgent problems and gained significant attention in recent years (Piao and Kim, 2016). In some regions with sensitive aquatic ecology in China, the total nitrogen (TN) concentration is limited to less than 10.0 mg L^–1^, which is superior to Chinese integrated wastewater discharge standard first-A (TN ≤ 15 mg L^–1^). However, the traditional secondary biological treatment was difficult to meet due to the strict TN discharge standard (Li et al., 2014). Hence, more and more advanced treatment technologies are needed to control TN discharge and protect the limited water sources.

Tertiary denitrification was required to further remove the nitrate nitrogen (NO_3_^–^-N) so as to achieve a high TN discharging standard since NO_3_^–^-N is the major component of TN in a secondary effluent. Denitrification deep-bed filter (DN-DBF) could transform NO_3_^–^-N to N_2_ and be considered to be an effective means to improve TN removal efficiency (Husband et al., 2014; Löwenberg et al., 2016; Shi et al., 2016). However, previous studies on DN-DBF just to meet one-class A discharge standard, for the parameters [such as carbon source type, chemical oxygen demand (COD) and TN (C/N) ratio, and hydraulic retention time (HRT)] of further advanced treatment were not yet clear (Cao et al., 2016; Xu et al., 2016).

Carbon source type and dosage play important roles that need to be optimized during the tertiary denitrification process for meeting a higher discharge standard. Many materials could be utilized as carbon sources, such as waste paper (Bao et al., 2016; Haske-Cornelius et al., 2021), biodegradable polymer (Wang et al., 2021), and wheat straw (Khokhar et al., 2014; Liu et al., 2021). Readily biodegradable organic matters are the optimal electron donors compared with refractory organic and other electron donors (Cao et al., 2016; Luo et al., 2020). Meanwhile, an insufficient dose would result in a low denitrification rate, while COD concentration would not meet the discharge standard when excessive dosage carbon sources are added (Ge et al., 2012). On the other hand, a conflict exists between TN removal efficiency and construction cost, which results from too long or too short HRT. Hence, appropriate C/N ratios and HRT should be optimized to achieve the optimum denitrification effect.

Meanwhile, microbial action in DN-DBF restricts the performance of TN removal, which indicates that it is necessary to analyze the characteristics of the microbial community in DN-DBF to reveal the mechanism of TN removal. Microbial communities and functional microorganisms in wastewater treatment are closely related to environmental and operational conditions (Adrados et al., 2014; Feng and Tong, 2015). Previous studies reported that the dominant denitrifying bacterial genera were *Dechloromonas*, *Acidovorax*, *Bosea*, *Polaromonas*, and *Chryseobacterium*, and particle sizes and packing type affected the community composition diversity (Tian and Wang, 2021; Yang et al., 2021). However, current studies only focused on the changes of microbial communities, but few on metabolic pathways.

Therefore, this study optimized operating parameters (carbon source type, C/N, and HRT) and investigated the performance of DN-DBF to ensure effluent compliance with the TN discharge standard (10.0 mg L^–1^). Besides, high-throughput sequencing technology was applied to reveal the degradation mechanism of pollutants.

## Materials and Methods

### Experimental Systems

**FIGURE 1:**
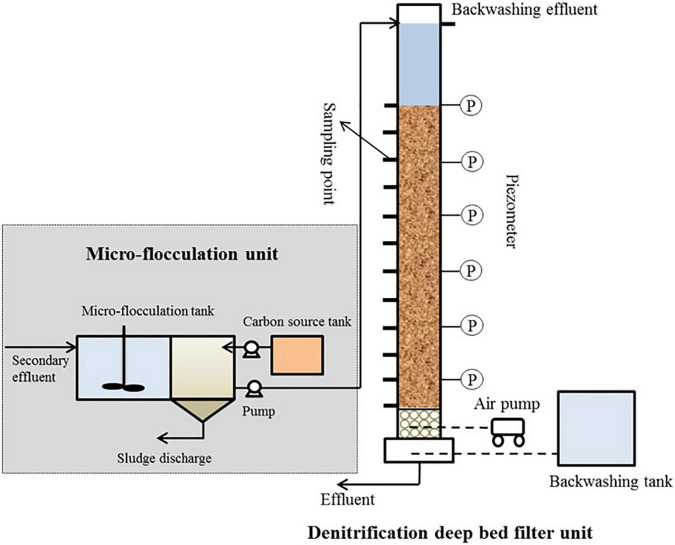
Schematic diagram of the denitrification deep bed filter.

The tertiary DN-DBF (Figure 1) was made from a plexiglass column with a diameter of 100 mm and a height of 1.8 m, and packed with 2–3 mm sizes of quartz sand. The packed height was 1.1 m, and a 0.3 m support gravel stone layer was set under that. The porosity and bulk density were 0.42 and 1.18 kg m^3^, respectively. The effluent of micro-coagulated was pumped to the top of DN-DBF and the effluent was discharged at the bottom. Besides, the sampling points and piezometers were installed on both sides of the DN-DBF and the intervals were 100 and 200 mm, respectively. The filtering media were backwashed every 24 h for 16 min by combined air and water for 8 min and water backwashing for 8 min. During backwashing, the air flow rate was 95.5 m h^–1^ and the water flow rate was 31.8 m h^–1^.

The secondary effluent was collected from an anaerobic-multistage anaerobic/oxic (A-MAO) process to a micro-flocculation tank, in which phosphorus was removed by polyaluminium chloride (PAC). The flocs were subsided in the settling tank, and the effluent was pumped into DN-DBF with external organic carbon. The main characteristics of the secondary effluent from the A-MAO process and the influent of DN-DBF are summarized in Table 1.

**TABLE 1:** Main characteristics of influent wastewater.

Parameter	Secondary effluent		Influent of DN-DBF	
	Range	Average	Range	Average
COD/(mg L^–1^)	15–40	28.6	40–100	–
NH_4_^+^-N/(mg L^–1^)	0–2	1.41	0–1.5	0.76
NO_3_^–^-N/(mg L^–1^)	7–11	8.51	7–11	8.62
TN/(mg L^–1^)	9–14	13.42	8–14	13.14
TP/(mg L^–1^)	0.30–0.50	0.43	0.10–0.30	0.18
pH	6.50–7.20	6.80	6.10–6.90	6.70
T/°C	22–30	27	22–30	27

### System Performance and Batch Experiments

Operating conditions of the DN-DBF are shown in Table 2. Carbon source type [methanol (CH_3_OH) and sodium acetate (NaAC)], C/N (7.0–8.0, 5.0–6.0, 4.0–5.0, 3.0–4.0, and 1.5–3.0), and HRT (0.5, 0.25, and 0.1 h) were investigated by long-time batch experiments at different periods. Under steady state, samples were taken at 0, 10, 30, 50, 70, 90, and 110 cm along the DN-DBF depth, and TN and COD concentrations were tested so as to investigate denitrification performance. Besides, the biomass attached on quartz sand samples and suspended biofilm were taken and measured from the DN-DBF regularly.

**TABLE 2:** Operation parameters of different stages.

Stages	Periods (day)	Operation parameters				
		Carbon source type	TN (mg L^–1^)	COD (mg L^–1^)	C/N	HRT (h)
I	1–10	Methanol (CH_3_OH)	13.15–14.78	39.87–45.93	3.0–3.5	0.25
II	10–20	Sodium acetate (NaAC)	13.05–14.61	39.56–42.95	3.0–3.5	0.25
III	20–29	CH_3_OH	12.42–15.04	99.0–110.98	7.0–8.0	0.5
IV	29–37	CH_3_OH	12.84–14.99	74.25–85.10	5.0–6.0	0.5
V	37–46	CH_3_OH	12.97–15.06	60.62–67.76	4.0–5.0	0.5
VI	49–55	CH_3_OH	13.15–14.78	39.87–46.32	3.0–4.0	0.5
VII	55–65	CH_3_OH	13.39–15.39	24.62–30.52	1.5–3.0	0.5
VIII	65–75	CH_3_OH	11.66–14.81	38.54–44.52	3.0–4.0	0.5
IX	75–85	CH_3_OH	11.72–14.52	39.41–45.29	3.0–4.0	0.25
X	85–95	CH_3_OH	12.65–15.62	40.12–46.55	3.0–4.0	0.1

### Chemical Analysis Methods

Samples were collected from the micro-flocculation setting tank and effluent of DN-DBF once a day. Besides, samples in different depths were collected when the system was in stable operation. COD, TN, ammonia nitrogen (NH_4_^+^-N), NO_3_^–^-N, and total phosphorus (TP) were analyzed according to standard methods (APHA, 2005). Biofilm biomass was determined using the weighted method. pH and temperature were monitored online by using WTW pH/Oxi 340i meter with dissolved oxygen (DO) and pH probes (WTW, Germany).

### Microbiological Analysis Methods

In order to evaluate the diversity of microbial community structure of the DN-DBF system, three biofilm samples were collected at the 95th day from three parts (10, 50, and 100 cm). The total DNA was extracted with the Fast DNA Spin Kit for Soil (MP Biomedicals, Santa Ana, CA, United States). The purified DNA was applied in polymerase chain reaction (PCR) analysis by PCR instrument (9700, GeneAmp^®^ ABI, Foster City, CA, United States) with the primer set of 338F (50-ACTCCTACGGGAGGCAGCA-30) and 806R (50-GGACTACCAGGGTATCTAAT-30). Besides, the PCR programs were as follows: initial denaturation at 95°C for 3 min; followed by 30 cycles of denaturation at 95°C for 30 s, annealing at 45°C for 30 s, elongation at 72°C for 45 s; and finally at 72°C for 10 min and 10°C until halted by user. The amplicons were sequenced on an Illumina MiSeq platform by Majorbio company (Shanghai, China). The original image data files were transformed into original sequencing sequence by CASAVA base recognition analysis, and the results were stored in FASTQ file format.

Paired-end reads of the original DNA fragments were merged using Trimmomatic and FLASH softwares (V1.2.7^1^) (Magoc and Salzberg, 2011), and sequencing reads were assigned to each sample based on a unique barcode. Mothur version v.1.30.1 was used to calculate microbial richness and diversity (ACE index, Chao 1 index, Simpson index, and Shannon index) (Schloss et al., 2011). The operational taxonomic units (OTUs) were assigned with Usearch software (version 7.1^2^), and all sequence column similarities within a stationary threshold (>97%) were combined together to be considered as one of the OTUs. Microbial abundance at the phylum and genus levels was counted depending on taxonomic data.

PICRUSt software^3^ was used to remove the 16S marker gene, and Non-supervised Orthologous Groups (eggNOG)^4^ databases and Kyoto Encyclopedia of Genes and Genomes (KEGG)^5^ were used to conduct the 16S rRNA functional prediction.

## Results and Discussion

### Optimization of Denitrification Deep-Bed Filter Operating Conditions

#### Carbon Source Types

**FIGURE 2:**
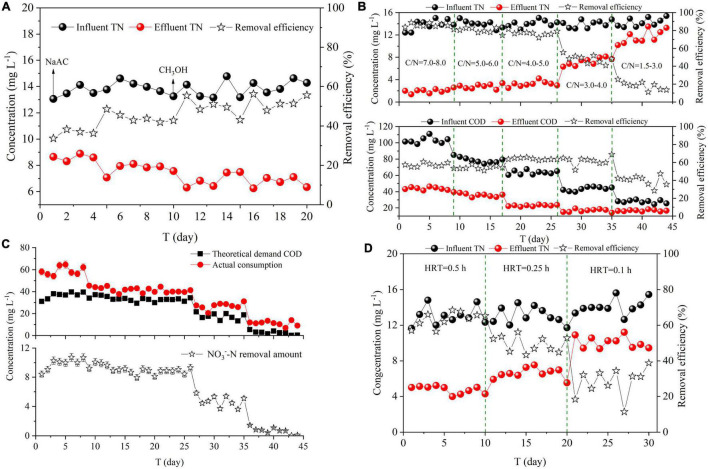
Effect of operating conditions on TN removal **(A)** Carbon source types, **(B,C)** C/N ratios, **(D)** HRT.

CH_3_OH and NaAC were added to DN-DBF. The effect of carbon source types on denitrification was compared, as shown in Figure 2A. The average TN removal efficiency was 41.11 and 51.04% with an effluent concentration of 8.09 and 6.80 mg L^–1^, respectively, when equal COD was dosed by CH_3_OH and NaAC. More 10% removal efficiency and 1.29 mg L^–1^ TN were removed by CH_3_OH than NaAC with 0.598 and 0.750 kg (m^3^ d)^–1^ TN translated into N_2_, respectively. Therefore, CH_3_OH was the optimal carbon source for denitrification of DN-DBF. The denitrification rate of using CH_3_OH as carbon source was higher than NaAC, even compared to previous studies in other bioreactor configurations (Table 3).

**TABLE 3:** Performance comparisons with other bioreactor configurations.

Carbon source	C/N ratio	Influent NO_3_^–^-N (mg L^–1^)	Denitrification rate [kg NO_3_^–^-N (m^3^ d)^–1^]	References
Sugar	6.25	40	3.21	Karanasios et al., 2016
Ethanol	1.5	9.8	0.001	Shi et al., 2016
No mention	10	1.5–2.0	0.082	Wang et al., 2016
Sodium acetate	3	14.5–19	0.343	Wu and Li, 2017
Brewery wastewater	5.2	30	1.11	Dong et al., 2012
Bakery wastewater	5.2	30	1.24	Dong et al., 2012
Methanol	5.2	30	1.44	Dong et al., 2012
Methanol	3.0–3.5	12–13	0.750	This study
Sodium acetate	3.0–3.5	12–13	0.598	This study

Due to the low cost and high efficiency of CH_3_OH and NaAC, they have been widely used by many sorts of denitrification process (Wei et al., 2016). CH_3_OH and NaAC requirement correlated with the removal of NO_3_^–^-N could be estimated by Eqs 1, 2; 2.47 g CH_3_OH (about 3.7 g COD) and 5.60 g CH_3_OONa (about 4.37 g COD) were consumed to transform 1 g NO_3_^–^ to N_2_. Therefore, more NaAC was consumed to achieve the denitrification process. Previous studies have shown that carbon source switchover resulted to the change of microbial community structure (Liang et al., 2014). Therefore, the difference in denitrification efficiency between methanol and sodium acetate may be caused by the difference in microbial community structure.


(1) $${\text{NO}}_{3}^{-}+1.08\,{\mathrm{CH}}_{3}\text{OH}+0.24\,H_{2}{\text{CO}}_{3}\to 0.06C_{5}{\text{H}}_{7}\,{\text{NO}}_{2}\,+0.47\,N_{2}+1.68\,H_{2}\text{O}+{\text{HCO}}_{3}^{-}$$

(2) $${\text{NO}}_{3}^{-}+1.06\,{\mathrm{CH}}_{3}{\text{OO}}^{-}+0.70\,H_{2}{\text{CO}}_{3}\to 0.15C_{5}{\text{H}}_{7}{\text{NO}}_{2}+0.42\,N_{2}+0.73\,H_{2}\text{O}+2.06\,{\mathrm{HCO}}_{3}^{-}$$

#### C/N Ratio

Five C/N ratios (7.0–8.0, 5.0–6.0, 4.0–5.0, 3.0–4.0, and 1.5–3.0) were examined to study the effect of their nitrogen removing performance on DN-DBF. As shown in Figure 2B, TN removal efficiency gradually decreased and the concentration in effluent rose as the C/N radio reduced. The removal efficiency of TN maintained stable vibration that ranged from 72.05 to 88.89% and effluent concentration was 1.38–3.54 mg L^–1^ when C/N ratio exceeded 4. With further reduction of C/N, stable TN removal performance was destroyed and the efficiency dropped to 13.65–25.33% with high TN concentration (10.20–15.39 mg L^–1^) in the effluent.

In addition, it should be noted that large-dosage carbon sources resulted in less TN concentration in the effluent, and excessive COD concentration would not meet the discharge standard, while an insufficient dose would lead to a low denitrification rate. COD in effluent was 21.36–46.32 mg L^–1^ at the first three periods (C/N was 7.0–8.0, 5.0–6.0, and 4.0–5.0). Though the COD value was inferior to discharge standard, the utilization of carbon source was low and more COD was wasted (Figure 2B). However, TN was over 10 mg L^–1^ with a low-concentration COD (15.51–18.32 mg L^–1^) when C/N was below 3.0. Hence, the optimal and economical influent C/N was 3.0–4.0, which could meet the TN discharge standard (10.0 mg L^–1^).

The theoretical demand of COD and actual consumption value is demonstrated in Figure 2C. The theoretical demand of COD was correlated by Eq. 1 and its less than actual consumption was due to the fact that partial carbon source was consumed or degraded by other processes instead of denitrification. Meanwhile, part of NO_3_^–^-N was absorbed for assimilation in the biological reactor (Constantin and Fick, 1997; Zhang et al., 2021). Cheng and Lin (1993) utilized methanol as a carbon source for denitrification and established the theoretical C/N ratio of 0.71. Wang et al. (2009) found that low C/N (C/N = 1) was not sufficient for the denitrification bacteria to grow. Nevertheless, Gómez et al. (2000), Fan et al. (2001), and Wei et al. (2016) concluded that the optimal C/N ratio was 1.25, 1.1, and 2.2 for denitrification with methanol as the electron donor, which was inconsistent with this study.

#### Hydraulic Retention Time

Hydraulic retention time, as another important parameter for DN-DBF, restricts the denitrification performance and the occupied area of structure. Figure 2D showed the effect of different HRT (0.5, 0.25, and 0.1 h) on TN removal. TN removal efficiency decreased and effluent concentration increased with the tightening of HRT, and the average TN removal efficiency was 63.37 and 49.47% with the effluent concentration of 4.75 and 6.59 mg L^–1^ when HRT was 0.5 and 0.25 h, respectively, while the steep increase of TN concentration in the effluent (average concentration was 10.08 mg L^–1^) reflected the fact that 0.1 h could not meet the discharge standard and the optimal and economical HRT was 0.25 h.

Denitrification is a rapid process of nitrogen conversion compared with the ammonia oxidation process. Farabegoli et al. (2003) drew a conclusion that 55% TN removal efficiency was obtained when influent TN concentration was 15 mg L^–1^ and HRT was 7 min. The above conclusion was similar to this study. Nevertheless, Shen and Wang (2011) used cross-linked starch/polycaprolactone blends as solid carbon source and concluded that the 26.86 mg NO_3_^–^-N (L h)^–1^ and 90% NO_3_^–^-N were removed at HRT 1 h, which was obviously more than those in this study. The phenomenon was inferred that carbon source style was the main factor that restricted HRT.

### Nutrient Removal and Head Loss Along the Filter Depth

**FIGURE 3:**
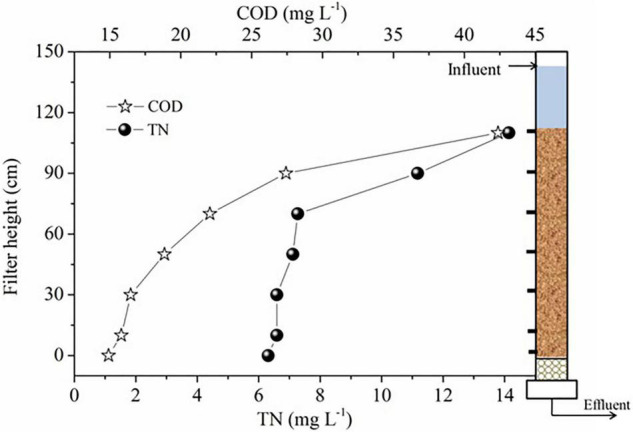
Concentration profiles of TN and COD along the DN-DBF depth.

Nutrient removal along the filter depth reflected the pollutants’ removal features of DN-DBF (Figure 3), the concentration of TN and COD in the influent were 14.14 and 42.37 mg L^–1^, and 55.37 and 64.81% removal efficiency were achieved with effluent concentration of 6.31 and 14.91 mg L^–1^. It is interesting that the denitrification rates of both pollutants were higher at the top of the filter than the bottom part; 48.85% TN and 47.93% COD were removed at the top 40 cm, while 5.52% TN and 16.87% COD were removed at the bottom 70 cm. The study of Dong et al. (2012) found that the specific denitrification rate along the bio-filter depth was 3.80, 1.21, 0.66, and 0.09 kg NO_3_^–^-N (m^3^ d)^–1^, respectively, and the highest specific denitrification rate appeared at 0–20 cm. It might be because more biomass accumulated on top of head loss; this is another standard to evaluate the performance of DN-DBF, and its change along the filter depth is shown in Table 4. As the increase of filter depth and operation time is extending, the head loss presented a significant increasing trend. The head loss changed by low increasing rates before the 12th hour, but the stable station was broken and the head loss jumped from 0.083–0.147 to 0.152–0.394 mH_2_O after the 12th hour. The specific incremental rate of head loss before the 12th hour along the filter depth was 0.007, 0.009, 0.010, 0.011, and 0.012 mH_2_O, respectively. However, the specific incremental rate after the 12th hour was 0.009–0.031 mH_2_O. Thus, the optimal backwash time was 12 h. Simate (2015) found that the head loss of the granular filter bed ran up suddenly at the 18th hour, which is similar to this study.

**TABLE 4:** Head loss along DN-DBF (m H_2_O).

Filter depth (cm)	*T* (h)							
	0	1	2	4	8	12	16	20
10	0.000	0.005	0.022	0.032	0.065	0.084	0.123	0.152
30	0.005	0.011	0.035	0.046	0.087	0.113	0.248	0.296
50	0.008	0.015	0.044	0.057	0.095	0.124	0.269	0.314
70	0.011	0.025	0.051	0.068	0.106	0.135	0.287	0.383
90	0.022	0.036	0.066	0.079	0.125	0.147	0.317	0.394

### Biofilm Biomass Along the Filter Depth

**FIGURE 4:**
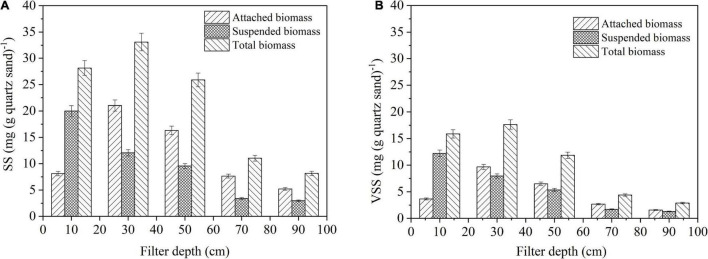
Change of biofilm biomass along the DN-DBF depth **(A)** SS and **(B)** VSS.

Biomass yield is an important indicator to be taken into account for explaining the denitrification performance of DN-DBF. Biomass yields in different filter depths are summarized in Figure 4; the total biomass and attached biomass increased at the first 30 cm of DN-DBF, and reduced as filter depth continued to increase. However, the suspended biomass was reduced along with the filter depth. The maximum quality of attached biomass, suspended biomass, and total biomass was 21.03, 20.01, and 33.10 mg SS (g quartz sand)^–1^, respectively, and appeared at 30, 10, and 30 cm. On the other hand, a similar rule was discovered for volatile suspended solids (VSS). The ratio of suspended solids (SS) and VSS was about 1.5–2.0, which indicated that approximately 40–50% inorganic particles exited and contributed to the head loss of DN-DBF.

At the top layer of this filter, the suspended biomass was more than the attached biomass, but it was opposite the other sampling point. The reason for this phenomenon may be that the SS was intercepted on the first 10 cm and hydraulic scour affected the formation of biofilms (Li et al., 2011; Wu et al., 2016). This result is consistent with Dong et al. (2012). There was a high correlation between biofilm biomass and effluent quality. Large TN and COD were removed at the top of the filter due to the accumulation of biofilm biomass. Meanwhile, the higher the biomass yield rates resulted from more COD and NO_3_^–^-N that were needed for complete denitrification and cell growth (Christensson et al., 1994).

### Microbial Mechanism of Nitrogen Removal

#### Microbial Richness and Diversity

The microbial biochemistry processes play an important role in the treatment of wastewater by DN-DBF. Therefore, analysis of the microbial process could help to characterize the mechanism and approach. In this section, 16S rRNA gene high-throughput sequencing technology was utilized to analyze the microbial community of the samples and assess the diversity and richness of the bacterial community, which was utilized to explain the denitrification mechanism of DN-DBF.

From 10 to 100 cm, the ACE and Chao index showed a tendency to rise and then fall, which indicated that the system owned the maximum microbial abundance when the filter depth was 50 cm (Table 5). Meanwhile, the variation of Shannon and Simpson diversity index from 10 to 100 cm illustrated that the diversity of microbial community also reached peak value at 50 cm. Community diversity was directly determined by species richness and species evenness. This phenomenon reflected that the backwashing process led to impacting on the stability of microorganisms and contributed to the lower diversity of microbial community than the middle part. Sun et al. (2016) and Huang et al. (2020) reported the maximal diversity index appeared at the middle part in the denitrification filler by comparing the diversity index (Shannon) along a denitrification filler, which was consistent with this study.

**TABLE 5:** Bacterial richness and diversity along DN-DBF.

0	ACE	Chao	Shannon	Simpson
10	1060	1059	50589	0.01017
50	1089	1094	5.707	0.00894
100	1064	1065	5.633	0.00886

#### Microbial Community Composition

**FIGURE 5:**
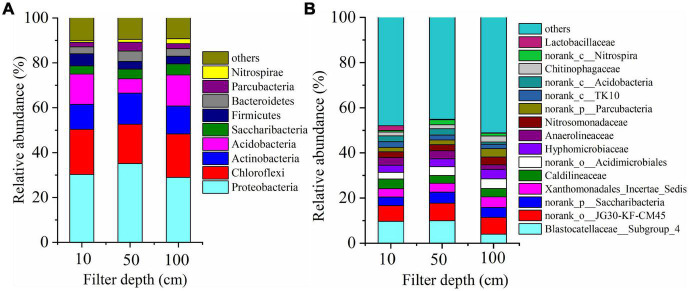
Change of microbial community composition along the DN-DBF depth **(A)** at phylum level and **(B)** at genus level.

During the process of operation, the bacteria with higher relative abundance of microbial community mainly affiliated to four phyla, i.e., Proteobacteria, Chloroflexi, Actinobacteria, and Saccharibacteria. All of them were substantiated to be the dominant denitrifying bacteria (Figure 5A). The results explained that the relative abundance of dominant denitrifying bacteria directly affected the denitrification efficiency. Previous researches have indicated that multiple types of Proteobacteria microorganisms were in several wastewater treatment bioreactors, which was in accord with the study of Ma et al. (2015). When the depth of filter was between 10 and 50 cm, Blastobacter was dominant and abundant, which was affiliated to α-Proteobacteria; it was a crucial participant in nitrogen removal and COD degradation in wastewater treatment.

Figure 5B demonstrates the dominant microbial flora abundance at family level. The abundance of microbial communities was evenly distributed, except for the *Blastocatellaceae_Subgroup_4* and *norank_o_JG30-KF-CM45* abundance of 6–10%, and the remaining flora abundance fluctuates between 2 and 5%. Chang et al. (2019) used a denitrification filter to remove polluted river, and results showed that *Blastocatellaceae* was the dominant heterotrophic denitrifying bacteria with a proportion of 2.3%. *JG30-KF-CM45* was also a typical denitrifying bacterium and proportional to the organic load in an urban sewage treatment process (Remmas et al., 2017). Meanwhile, the *norank_p_Saccharibacteria* under the Spirulina was reported as a denitrification group participating in organic matter removal (Sun et al., 2018). *Caldilineaceae* was with an abundance of about 4%, which was similar to previous reports such as Wu et al. (2018) who found that its share in short-range denitrification was 3.72%. Besides, *Xanthomonadales_Incertal_Sidis*, *Hyphomicrobiaceae*, and *norank_p_Parcubacteria* are typical heterotrophic anti-nitrifying bacteria. *norank_c_Acidobacteria*, *Lactobacilliaceae*, and *norank_o_Acidimicrobiales* were organic degradation bacteria belonging to Acidobacteria, which was related to the addition of carbon sources in the influent. *Nitrosomonadaceae* and *norank_c_Nitrospira* are nitrifying bacteria, whose appearance indicated that nitrification occurred at the upper and end of this filter.

#### Pathway of Nitrogen Metabolism

**FIGURE 6:**
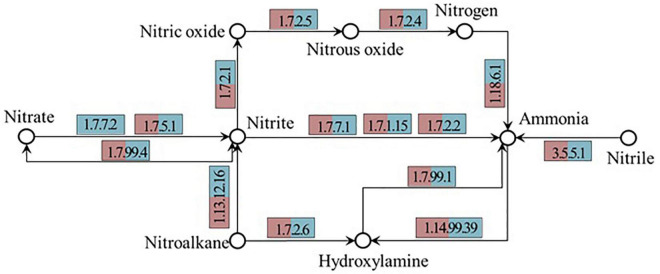
Nitrogen cycle related key enzymes along the DN-DBF depth.

Figure 6 and Table 6 demonstrate the abundance of key enzymes related to nitrogen cycle depending on functional prediction. Specific enzymes, such as nitrate reductase (EC1.7.2.1), chlorophyllide reductase iron protein subunit X (EC1.18.6.1), nitronate monooxygenase (EC1.13.12.16), and nitrilase (EC3.5.5.1), are involved in the denitrification process of DN-DBF. With the augmentation of the depth of the filter, the reduction of chlorophyllide reductase iron protein subunit X (EC1.18.6.1), nitrilase (EC3.5.5.1), nitrate reductase 2 (EC1.7.99.4), nitric oxide reductase (EC1.7.2.5), and chlorophyllide reductase iron protein subunit X (EC1.18.6.1) took on an upward and then downward tendency. Huang et al. (2020) discussed the abundance of key enzymes related to nitrogen cycle in an iron carbon-based constructed wetland (CW), and chlorophyllide reductase iron protein subunit X (EC1.18.6.1) in CW with Fe^0^-C filter demonstrated the lowest abundance at the middle layer and the highest value appeared in CW with ceramsite filler, which was consistent with this study.

**TABLE 6:** Abundance of nitrogen cycle-related key enzymes along DN-DBF (10^–3^ %).

Enzyme	10 cm	50 cm	100 cm	Function description
EC1.7.99.4	58.47	66.99	66.51	Nitrate reductase 2, delta subunit
EC1.7.2.1	4.06	5.18	5.92	Nitrite reductase (NO-forming)
EC1.7.2.5	5.50	5.97	4.94	Nitric oxide reductase, cytochrome *b*-containing subunit I
EC1.7.2.4	17.83	16.88	15.74	Nitrous-oxide reductase
EC1.7.7.1	13.00	10.56	13.50	Ferredoxin-nitrite reductase
EC1.7.2.2	7.88	7.49	8.94	Formate-dependent nitrite reductase, periplasmic cytochrome c552 subunit
EC1.18.6.1	86.08	105.08	96.97	Chlorophyllide reductase iron protein subunit X
EC3.5.5.1	41.60	48.91	40.71	Nitrilase
EC1.7.99.1	1.31	1.37	1.32	Hydroxylamine reductase
EC1.13.12.16	25.52	23.59	24.32	Nitronate monooxygenase

At the filter depth of 50 cm, the abundance of relative enzyme reached the maximum, and the number and denitrifying activity of corresponding denitrifying bacteria was the highest; therefore, the denitrification was the strongest with the highest nitrogen removal rate in this part. The results coincided with the changes of microbial community composition and diversity. During the denitrification process, nitrite reductase controlled the reduction of NO_3_^–^-N and the production of N_2_O, and the process of transforming N_2_O to NO was commanded by nitrous oxide reductase. The relative abundance of nitrite reductase gradually increases as the depth increases, and conversely, the relative abundance of nitrous oxide reductase gradually decreased. It indicated that more N_2_O is converted to NO with the deepening of the filter. It is worth noting that the relative abundance of nitronate monooxygenase demonstrated a decrease first and then ascended. In view of the effect of influent and backwashing, DO was enriched on both ends of the filter. Hence, the abundance of aerobic bacteria was relatively large and resulted in high nitronate monooxygenase abundance in the center of this filter.

## Conclusion

The DN-DBF parameters were optimized for the advanced treatment of TN from the secondary effluent. More than 12.7% of TN removal efficiency was obtained by adding methanol compared with sodium acetate, and the optimal influent C/N ratio and HRT were 3:1 and 0.25 h, respectively. The backwash time was 12 h, and the total and attached biomass reached the maximum at 30 cm of DN-DBF, where the diversity and richness of microbial community was higher than that in both ends. High-throughput sequencing technology showed that Proteobacteria, Chloroflexi, Actinobacteria, and Saccharibacteria were dominant flora at phylum level, and *Blastocatellaceae_Subgroup_4 and norank_o_JG30-KF-CM45* were dominant denitrification floras with an abundance of 6–10%. Meanwhile, the abundance of nitrite reductase enzymes (EC1.7.2.1) reached the maximum at 50 cm.

## Data Availability Statement

The original contributions presented in the study are included in the article/supplementary material, further inquiries can be directed to the corresponding authors.

## Author Contributions

ZD and TC made substantial contributions to the conception or design of the work and the acquisition, analysis, or interpretation of data for the work, drafted the work or revised it critically for important intellectual content, gave final approval of the version to be published, and agreed to be accountable for all aspects of the work in ensuring that questions related to the accuracy or integrity of any part of the work are appropriately investigated and resolved. All authors listed have made a substantial, direct, and intellectual contribution to the work, and approved it for publication.

## Conflict of Interest

The authors declare that the research was conducted in the absence of any commercial or financial relationships that could be construed as a potential conflict of interest.

## Publisher’s Note

All claims expressed in this article are solely those of the authors and do not necessarily represent those of their affiliated organizations, or those of the publisher, the editors and the reviewers. Any product that may be evaluated in this article, or claim that may be made by its manufacturer, is not guaranteed or endorsed by the publisher.

## Footnotes

1
http://ccb.jhu.edu/software/FLASH/

2
http://drive5.com/uparse/

3
http://huttenhower.sph.harvard.edu/galaxy/root?tool_id=PICRUSt_normalize/

4
http://eggnog.embl.de/

5
http://www.genome.jp/kegg/
